# National Survey of Point-of-Care Ultrasound Scholarly Tracks in Emergency Medicine Residency Programs

**DOI:** 10.5811/westjem.2021.5.52118

**Published:** 2021-08-21

**Authors:** Stephen Alerhand, Elaine Situ-Lacasse, Christine Ramdin, Michael Gottlieb

**Affiliations:** * Rutgers New Jersey Medical School, Department of Emergency Medicine, Newark, New Jersey; † University of Arizona College of Medicine, Department of Emergency Medicine, Phoenix, Arizona; ‡ Rush University Medical Center, Department of Emergency Medicine, Chicago, Illinois

## Abstract

**Introduction:**

Residency scholarly tracks are educational programs, designed to help trainees develop an area of expertise. Although the breadth of residency point-of-care ultrasound (POCUS) education has developed considerably in recent years, there is no literature to date describing scholarly tracks specifically in POCUS. In this study we sought to determine the prevalence, characteristics, and outcomes of POCUS scholarly tracks in emergency medicine (EM).

**Methods:**

This was a cross-sectional survey of EM residency programs accredited by the Accreditation Council for Graduate Medical Education. Surveys were distributed between March–August 2020 using a listserv followed by targeted emails to residency and ultrasound leadership. We summarized data using descriptive statistics, and performed logistic regression to identify factors associated with a POCUS scholarly track.

**Results:**

Of 267 residency programs 199 (74.5%) completed the survey. Fifty-seven (28.6%) had a POCUS scholarly track as of the 2019–2020 academic year. Scholarly tracks in POCUS were more common in university-based/academic sites and larger residency programs. Of the 57 programs with POCUS scholarly tracks, 48 (84.2%) required residents to present at least one POCUS lecture, 45 (78.9%) required residents to serve as instructor at a hands-on workshop, and 42 (73.7%) required residents to participate in quality assurance of departmental POCUS scans. Only 28 (49.1%) tracks had a structured curriculum, and 26 (45.6%) required POCUS research. In total, 300 EM residents completed a POCUS scholarly track over the past three academic years, with a median of 4 (2–9) per program. Seventy-five (25.0%) proceeded to a clinical ultrasound fellowship after residency graduation, with a median of 1 (interquartile range 0–2) per program. A total of 139 POCUS-specific abstracts (median 2 [0–3]) and 80 peer-reviewed manuscripts (median 1 [0–2]) were published by scholarly track residents over the past three years.

**Conclusion:**

This survey study describes the current prevalence, characteristics, and outcomes of POCUS scholarly tracks across EM residency programs. The results may inform the decisions of residency programs to create these tracks.

## BACKGROUND

Scholarly tracks in emergency medicine (EM) are educational programs or curricula within residency programs designed to help trainees develop a focused area of expertise.^1^ A 2017 survey found that the perceived benefits of scholarly tracks included advanced training (92%), career guidance (88%), mentorship (88%), and preparation for an academic career (80%).^2^ Residency programs with tracks were also more likely to graduate residents to an academic career.^2^ A 2018 search of residency program websites found that 33 (21.2%) of 156 programs had some form of scholarly track, although this data was limited to general tracks.^3^

As the use of point-of-care ultrasound (POCUS) has expanded in EM, so has the breadth of residency POCUS education. A 2003 survey of POCUS training in EM residency programs found diverse curricular implementation.^4^ A subsequent 2010 survey found a discrepancy between EM residency programs’ POCUS curricula and perceived needs for proficiency.^5^ In 2016, however, the American College of Emergency Physicians (ACEP) published a policy statement delineating the EM scope of POCUS practice, learning objectives, and recommendations for residency POCUS education.^6^ By 2017, 88% of programs had a dedicated POCUS rotation.^7^ The desire and need for advanced training have also expanded, as evidenced by a 240% increase in clinical ultrasound fellowship graduates between 2009–2019 (R. Gaspari, personal communication, December 1, 2020).

Despite the increasing interest in POCUS, no literature to date specifically describes scholarly tracks in POCUS. Their existence, individual characteristics, and standardization across EM residency programs remain unclear. To develop best practices in POCUS education and prepare residents for fellowship and academic careers, the current practice must first be understood. In this study we sought to assess the prevalence, characteristics, and outcomes of POCUS scholarly tracks in EM residency programs.

## METHODS

### Study Setting and Participants

We compiled a list of all EM residency programs accredited by the Accreditation Council for Graduate Medical Education on March 1, 2020 (https://apps.acgme.org/ads/Public/Reports/Report/1). All programs identified via this list were eligible for participation. Their geographic regions were defined according to the Society of Academic Emergency Medicine regional meeting designations (https://www.saem.org/docs/default-source/membership/2020-regionalmtgapplic_current-revised-11-15-2019.pdf?sfvrsn=67fc01fd_0). We collected data from surveys completed between March–August 2020. The study was approved by the institutional review board at the Rutgers New Jersey Medical School.

### Study Design

This was a cross-sectional survey study. We used several methods to contact the programs, as guided by the modified Dillman methodology.^8^ First, we sent the survey through the ACEP Ultrasound Section listserv. Then, for all programs that had not yet responded, we sent individualized emails to the ultrasound director, ultrasound fellowship director, residency program director, and associate residency program director. We emailed reminders one month and two months later to non-responders. In cases where the survey was completed by multiple respondents from the same institution, we only analyzed the data from one survey by prioritizing responses in the following order: ultrasound director, ultrasound fellowship director, residency program director, ultrasound resident education director, associate residency program director, ultrasound undergraduate medical education director, ultrasound research director, and other ultrasound faculty. Study data were collected and managed using Research Electronic Data Capture (REDCap, Vanderbilt University, Nashville, TN).

**Population Health Research Capsule**

What do we already know about this issue?

*Despite the breadth of residency point-of-care ultrasound (POCUS) education, no literature to date describes scholarly tracks specifically in POCUS*.

What was the research question?

*We sought to determine the prevalence, characteristics, and outcomes of POCUS scholarly tracks across EM residencies*.

What was the major finding of the study?

*Scholarly tracks in POCUS were present in 29% of programs and included variation in training components*.

How does this improve population health?

*The results may inform the decisions of ultrasound directors and residency program directors when considering the creation of POCUS scholarly tracks*.

### Survey Development

We designed the surveys in accordance with best practices in survey design.^9^ The initial questions were developed based upon a literature review and experience as directors of POCUS programs. We then sought additional input from other ultrasound educators. The survey was iteratively refined as a group. Then the survey was piloted with in-person feedback from residency program leaders and ultrasound division directors from various institutions. The survey was modified in accordance with this feedback (Appendix A).

### Statistical Analysis

We summarized data using descriptive statistics, including proportions and either means with 95% confidence intervals (CI) or medians with interquartile ranges (IQR), depending upon the normality of the data. Data were categorized and tested for normality using the Shapiro-Wilk test. To determine what program characteristics were best associated with that program having a POCUS scholarly track, we used a binomial logistic regression. Categories with fewer than five responses were excluded. We used Mann-Whitney U tests to determine whether there was a relationship between having a research requirement and the number of POCUS-related abstracts and publications generated by the program. All *P*-values were reported at a significance level of 0.05.

## RESULTS

There were 267 potentially eligible residency programs at the time of the study. After removal of duplicate responses (ie, multiple respondents from the same institution), 199 (74.5%) unique programs completed the survey (Table 1). More than half (53.8%) of surveys analyzed were completed by ultrasound division directors (Appendix B). Of programs that responded to the survey, 57 (28.6%) had a POCUS scholarly track as of the end of the 2019–2020 academic year. Using a binomial logistic regression, we found that characteristics associated with residencies having a POCUS scholarly track included the following: self-defining as a university-based/academic site (odds ratio [OR] 5.32; 95% CI, 1.29–22.00]; and having a larger number of residents in the program (OR 1.04; 95% CI, 1.01–1.06) (Table 2).

Among the 142 (71.4%) programs that did not have a POCUS scholarly track, the most indicated reason was that there were no scholarly tracks in the residency at all (n = 77, 54.2%) (Table 3). Out of those programs, 25 (17.6%) indicated that they planned to have a POCUS scholarly track for the upcoming academic year. Of all 199 survey respondents, 114 (57.3%) indicated interest in receiving guidance on development of a POCUS scholarly track.

Of the 57 programs with POCUS scholarly tracks, 48 (84.2%) required residents to present at least one POCUS lecture, 45 (78.9%) required residents to serve as an instructor at a hands-on workshop, and 42 (73.7%) required residents to participate in quality assurance of departmental POCUS images (Table 4). Only 28 (49.1%) tracks had a structured curriculum, and 26 (45.6%) required POCUS research.

From the programs offering POCUS scholarly tracks, 300 total EM residents completed the track over the past three academic years, with a median of four (IQR 2–9) per program (Table 5). Of these 300 residents, 75 (25.0%) proceeded to a clinical ultrasound fellowship after residency graduation, with a median of 1 (IQR 0–2) per program. A total of 139 POCUS-specific abstracts were presented at academic conferences over the past three years by residents completing a POCUS scholarly track, with a median of two (IQR 0–3) per program. Over this time, a total of 80 POCUS-specific, peer-reviewed publications were generated, with a median of 1 (IQR 0–2) per program. Among programs with a track, having a research requirement did not have an effect on the number of abstracts (*P* = 0.896) or publications generated (*P* = 1.000).

Of programs with dedicated elective time to pursue track goals (n = 30; 52.6%), the length of dedicated time varied by program. Eleven (36.7%) programs provided their residents with > eight weeks, five (16.7%) programs provided six weeks, 8 (26.7%) programs provided four weeks, four (13.3%) programs provided two weeks, and two (6.7%) programs provided one week.

## DISCUSSION

Our study is the first to assess the prevalence, characteristics, and outcomes of POCUS scholarly tracks across United States EM residency programs. We found that only 28.6% of responding programs had a POCUS scholarly track. This is slightly higher than the 2018 online search study in which 21.2% programs offered any type of scholarly track.^3^ The most common reason provided for not having a POCUS track was that the residency had no tracks at all. Thus, this may reflect an issue not specific to POCUS but rather to all scholarly tracks in general.

As might be expected, university-based/academic residency programs were more likely to have a POCUS scholarly track. Larger residency sizes were also more likely to have a POCUS scholarly track, which is consistent with the 2017 survey describing general scholarly tracks.^2^ In contrast, the duration of residency did not affect whether a program had a POCUS track. This differs from both the 2017 survey^2^ and 2018 online search study,^3^ both of which found higher rates in four-year programs. This result was surprising, as many four-year programs specifically advertise the extra year as an opportunity to develop a focused academic niche. Considering that clinical ultrasound fellowship programs have a positive impact on residents’ POCUS educational experiences,^10^ it was also surprising that neither the number of clinical ultrasound fellowship-trained faculty nor the presence of a clinical ultrasound fellowship itself had an association with a residency offering a POCUS track. For programs without a POCUS track, the most significant contributing factor was the lack of scholarly tracks in general, whereas only 25.4% reported insufficient faculty availability and 15.5% reported inadequate faculty expertise as reasons. This differs from the 2017 survey of general scholarly tracks, in which the most common reason reported for not having tracks was insufficient faculty.^2^

Among the 57 residencies with POCUS scholarly tracks, there was significant variation in the individual components of these tracks. This is consistent with the 2003 survey finding diverse implementations of residency POCUS curricula.^4^ This variation in track components may reflect the wide range of interests, backgrounds, and resources of POCUS faculty across EM residency programs. Future research should determine which components are most valuable for learners, in order to guide programs seeking to create or improve existing scholarly tracks.

In total, POCUS scholarly tracks led to 139 POCUS-specific abstracts and 80 POCUS-specific, peer-reviewed publications over the past three years by scholarly track residents. Scholarly tracks provide an opportunity for residents to gain experience with research and meet their residency scholarly requirement. A review of published research abstracts at the Society of Academic Emergency Medicine Annual Meeting found that from 1999–2015, there was a 10.2% increase in the number of accepted abstracts related to POCUS research, with a 26.6% increase in the number of unique authors.^11^ While our study did not find an association between a specific research requirement and abstracts or publications, a required research component to the track may still be of interest to residency program directors and ultrasound division directors looking to increase their department’s POCUS scholarly output.

Despite the lower number of programs with a POCUS scholarly track, almost one-fifth of residency programs without a POCUS scholarly track responded that they would be developing one over the upcoming academic year. Over half of all total respondents also expressed interest in receiving guidance for developing a POCUS track. The only published model to date of a POCUS scholarly track describes a single program and may thus not be applicable to all EM residencies.^12^ A 2009 academic working group discussed general scholarly tracks and made the following recommendations for fostering successful implementation: creating clear goals and objectives for each track; matching track topics with faculty expertise; protecting time for both faculty and residents; providing adequate mentorship for residents; publicizing accomplishments internally and monitoring progress; and refining the tracks regularly.^1^ We found that 49.1% of POCUS scholarly tracks consisted of a structured curriculum toward meeting goals or milestones, and 52.6% of tracks provided dedicated time to pursue track goals. Therefore, there is a need and desire to develop best practice consensus guidelines offering strategies for developing and sustaining successful POCUS residency scholarly tracks.

## LIMITATIONS

The results of our survey study are subject to the limitations inherent to this form of data collection. For instance, there may have been selection bias toward those programs with POCUS tracks. However, we were able to achieve a response from three-quarters of programs by using serial surveys delivered through multiple distribution methods, leading to a higher response rate than the 2017 online survey of general scholarly tracks.^2^ In addition, as a cross-sectional study, only one time period was evaluated. The survey results may change as more EM residencies are created and POCUS continues to advance as a subspecialty. Thirdly, since we did not track the effectiveness of individual program components, we were unable to comment on which components are the most valuable to have in a POCUS scholarly track.

We also did not compare the academic rigor or scholarly output between POCUS scholarly track residents and “POCUS-interested” residents in those programs without a POCUS scholarly track, as the standard or criteria for what constituted a “POCUS-interested” resident would vary widely among survey respondents from different types of residency programs. Finally, it is possible that some programs may have educational programs or curricula that may not be defined as scholarly tracks but share some overlap with scholarly tracks. While we asked programs to self-identify scholarly tracks based on the definition, some programs may not have self-identified in that manner, leading to potential under-reporting in those programs.

## CONCLUSION

This study describes the current prevalence, characteristics, and outcomes of POCUS scholarly tracks across United States EM residency programs. The results of this study may inform the decisions of ultrasound division directors and residency program directors when considering the creation of scholarly tracks in their own programs. The broad interest in receiving guidance on POCUS scholarly tracks also lends support to the future development of expert consensus guidelines.

## Supplementary Information





## Figures and Tables

**Table 1:** Demographics of responding residency programs (n = 199).

Demographic variable	Number of respondents (%)
Region	
Great Plains (IA, IL, KS, MO, MN, NE, ND, SD, WI)	19 (9.5%)
Mid-Atlantic (DC, DE, MD, NJ, NY, PA, VA)	58 (29.1%)
Midwest (IN, KY, MI, OH, WV, IN)	30 (15.1%)
New England (CT, MA, ME, NH, RI, VT)	12 (6.0%)
South Central (AR, LA, OK, TX)	18 (9.0%)
Southeastern (AL, FL, GA, MS, NC, PR, SC, TN)	32 (16.1%)
Western (AK, AZ, CA, CO, HI, ID, NM, NT, NV, OR, UT, WA, WY)	30 (15.1%)
3-year residency	148 (74.4%)
4-year residency	51 (25.6%)
Number of residents (median [Q1–Q3])	33 [24–48]
Category of primary residency site*	
University-based/academic	116 (58.3%)
Non-university-based	67 (33.7%)
County/public hospital	29 (14.6%)
Military	4 (2.0%)
Other	3 (1.5%)
Number with an ultrasound image archiving system	142 (71.2%)
Number of clinical ultrasound fellowship-trained faculty (median [Q1–Q3])	3 [1–4]
Number with an associated clinical ultrasound fellowship	105 (52.8%)

* Responders were allowed to select more than one type of clinical site

**Table 2:** Factors associated with residency programs having a point-of-care ultrasound scholarly track (n = 57).

Variable	Odds ratio (95% Confidence Interval)
University-based/academic site+	5.32 (1.29–22.00)*
Non-university-based site+	3.55 (0.79 – 15.93)
County/public hospital+	2.71 (0.88 – 8.35)
4-year residency	1.70 (0.75 – 3.85)
3-year residency	0.59 (0.26 – 1.34)
Number of clinical ultrasound fellowship-trained faculty	1.05 (0.88 – 1.24)~
Number of residents	1.04 (1.01 – 1.06)*~
Program has an associated clinical ultrasound fellowship	1.05 (0.44 – 2.55)

* Statistically significant.

+ Responders were allowed to select more than one type of clinical site.

~ Per additional faculty and resident, respectively.

**Table 3:** Reasons provided for residency programs not having a point-of-care ultrasound scholarly track (n = 142).

Reasons*	Number of respondents (%)
No scholarly tracks in the residency at all	77 (54.2%)
Insufficient faculty availability	36 (25.4%)
Insufficient faculty expertise	22 (15.5%)
Redundancy with other residency activities	22 (15.5%)
Insufficient time within resident schedule	19 (13.4%)
Insufficient funding	16 (11.3%)
Insufficient resident interest	15 (10.6%)
Program director preference	7 (4.9%)
Effort to maintain outweighs the products	6 (4.2%)
Trial was unsuccessful	1 (0.7%)
Chair preference	0 (0%)
Other	21 (14.8%)

* Responders were allowed to select more than one reason.

**Table 4:** Characteristics of existing point-of-care ultrasound scholarly tracks (n = 57).

Characteristics	Yes (%)	No (%)	Not sure (%)
Clinical			
Required to perform a certain threshold number of POCUS scans?	30 (52.6%)	22 (38.6%)	5 (8.8%)
Required to complete an advanced POCUS elective (eg, regional anesthesia, transesophageal echocardiography)?	20 (35.1%)	34 (59.6%)	3 (5.3%)
Structured curriculum toward meeting goals or milestones?	28 (49.1%)	26 (45.6%)	3 (5.3%)
Education			
Required to present a POCUS lecture to students, residents, and/or faculty?	48 (84.2%)	8 (14.0%)	1 (1.8%)
Required to serve as hands-on instructor at a POCUS workshop?	45 (78.9%)	10 (17.5%)	2 (3.5%)
Administration			
Required to participate in quality assurance of emergency department POCUS scans?	42 (73.7%)	13 (22.8%)	2 (3.5%)
Required to participate in a POCUS-focused quality improvement project?	28 (49.1%)	27 (47.4%)	2 (3.5%)
Research and Scholarly Activity			
Required to conduct POCUS-focused research?	26 (45.6%)	29 (50.9%)	2 (3.5%)
Required to attend a POCUS-focused conference?	23 (40.4%)	31 (54.4%)	3 (5.3%)
Required to present a POCUS-focused abstract at an ultrasound or emergency medicine conference?	12 (21.1%)	42 (73.7%)	3 (5.3%)
Required to contribute to a POCUS-focused manuscript in a peer-reviewed journal publication?	8 (14.0%)	45 (78.9%)	4 (6.9%)
Dedicated non-clinical time to pursue scholarly track goals?	33 (57.9%)	22 (38.6%)	2 (3.4%)

*POCUS*, point-of-care ultrasound.

**Table 5:** Outcomes of point-of-care ultrasound scholarly tracks over the past three academic years (n = 57).

Outcome	Median (IQR)	Range	Total
Number of graduates	4 (2–9)	1–25	300
Number of graduates who proceeded to clinical ultrasound fellowship	1 (0–2)	0–4	75
Number of abstracts presented externally at conferences	2 (0–3)	0–20	139
Number of peer-reviewed publications generated	1 (0–2)	0–10	80

*IQR*, interquartile range.

## References

[1] Regan L, Stahmer S, Nyce A (2010). Scholarly tracks in emergency medicine. Acad Emerg Med.

[2] Jordan J, Hwang M, Kaji AH (2018). Scholarly tracks in emergency medicine residency programs are associated with increased choice of academic career. West J Emerg Med.

[3] Jordan J, Hwang M, Coates WC (2018). Academic career preparation for residents: Are we on the right track? Prevalence of specialized tracks in emergency medicine training programs. BMC Med Educ.

[4] Counselman FL, Sanders A, Slovis CM (2003). The status of bedside ultrasonography training in emergency medicine residency programs. Acad Emerg Med.

[5] Ahern M, Mallin MP, Weitzel S (2010). Variability in ultrasound education among emergency medicine residencies. West J Emerg Med.

[6] (2017). Ultrasound Guidelines. Emergency, point-of-care and clinical ultrasound guidelines in medicine. Ann Emerg Med.

[7] Gottlieb M, Arno K, Kuhns M (2018). Distribution of clinical rotations among emergency medicine residency programs in the United States. AEM Educ Train.

[8] Hoddinott SN, Bass MJ (1986). The Dillman total design survey method. Can Fam Physician.

[9] Artino AR, La Rochelle JS, Dezee KJ (2014). Developing questionnaires for educational research: AMEE Guide No. 87. Med Teach.

[10] Adhikari S, Raio C, Morrison D (2014). Do emergency ultrasound fellowship programs impact emergency medicine residents’ ultrasound education?. J Ultrasound Med.

[11] Prats MI, Bahner DP, Panchal AR (2018). Documenting the growth of ultrasound research in emergency medicine through a bibliometric analysis of accepted academic conference abstracts. J Ultrasound Med.

[12] Boulger C, Adams DZ, Hughes D (2017). Longitudinal ultrasound education track curriculum implemented within an emergency medicine residency program. J Ultrasound Med.

