# Supplementary material for: National Survey of Point-of-Care Ultrasound Scholarly Tracks in Emergency Medicine Residency Programs
Source: West J Emerg Med. 2021 Aug 21;22(5):1095–101. doi: 10.5811/westjem.2021.5.52118 (PMC8463042; doi:10.5811/westjem.2021.5.52118)
Supplement: Supplementary file 1 [file wjem-22-1095-s001.docx]

**Appendix A: Survey Distributed to Eligible Respondents**

| **DEMOGRAPHICS** | | | | | | | | | |
| --- | --- | --- | --- | --- | --- | --- | --- | --- | --- |
| 1. Which state is your residency in? | *AK AL AR AZ CA CO CT DC DE FL GA HI IA ID IL IN KS KY LA MA MD ME MI MN MO MS MT NC ND NE NH NJ NM NV NY OH OK OR PA PR RI SC TN TX UT VA VT WA WI WV WY* | | | | | | | | |
| 1. What is your designated position (select all that apply)? | - *Ultrasound Division Director* - *Fellowship Director* - *Ultrasound Resident Education Director* - *Ultrasound Undergraduate Medical Education Director* - *Ultrasound Research Director* - *Other Ultrasound Faculty* - *Residency Program Director* - *Residency Associate Program Director* | | | | | | | | |
| 1. Is your residency a 3- or 4-year program (as of 2019-2020 academic year)? | *3* | | | | | *4* | | | |
| 1. How many total residents are in your program (as of 2019-2020 academic year)? |  | | | | | | | | |
| 1. Which category is your primary residency site (select all that apply)? | - *University-based/academic* - *Non-university-based* - *County/public hospital* - *Military* - *Other* | | | | | | | | |
| 1. Does your department have an ultrasound image archiving system for quality assurance (e.g. Q-Path, Ultralinq, Softlink) (as of 2019-2020 academic year)? | *Yes* | | | | *No* | | | | |
| 1. How many Clinical Ultrasound fellowship-trained faculty are in your department (as of 2019-2020 academic year)? |  | | | | | | | | |
| 1. Does your department have a Clinical Ultrasound fellowship (as of 2019-2020 academic year)? | *Yes* | | | | *No* | | | | |
| 1. Does your residency have a POCUS Scholarly Track (as of 2019-2020 academic year)? | *Yes* | | | | *No* | | | | |
| If yes, how many graduating residents have participated over the past 3 years (graduating classes of 2018, 2019, and 2020)? |  | | | | | | | | |
| If yes, how many POCUS Scholarly Track residents have matched into a Clinical Ultrasound fellowship over the past 3 years (graduating classes of 2018, 2019, and 2020)? |  | | | | | | | | |
| If no, what are the reasons (select all that apply)? | - *Insufficient faculty expertise* - *Insufficient faculty availability* - *Insufficient resident interest* - *Insufficient time within resident schedule* - *Program Director preference* - *Chair preference* - *Insufficient funding* - *Redundancy with other residency activities* - *Effort to maintain outweighs the products* - *Trial was unsuccessful* - *No scholarly tracks in the residency at all* - *Other (please specify)* | | | | | | | | |
| If no, do you plan to develop a POCUS Scholarly Track for the upcoming academic year? | *Yes* | | *No* | | | | *Don’t know/Not sure* | | |
| 1. Would you be interested in receiving guidance on developing a POCUS Scholarly Track? | *Yes* | | *No* | | | | *Don’t know/Not sure* | | |
| **CHARACTERISTICS OF POCUS SCHOLARLY TRACK (if applicable)** | | | | | | | | |  |
| **Clinical** | | | | | | | | |  |
| 1. Beyond the ACGME requirement (150 scans), is there a requirement for the number of scans performed by scholarly track residents prior to graduation? | | *Yes* | | *No* | | | | *Don’t know/Not sure* |  |
| If yes, how many scans are required? | |  | | | | | | |  |
| 1. Are scholarly track residents required to complete an advanced POCUS elective (e.g. transesophageal echocardiography, regional anesthesia)? | | *Yes* | | *No* | | | | *Don’t know/Not sure* |  |
| 1. Is there a structured curriculum for scholarly track residents geared toward meeting milestones? | | *Yes* | | *No* | | | | *Don’t know/Not sure* |  |
| **Education** | | | | | | | | |  |
| 1. Are scholarly track residents required to present a POCUS lecture to students, residents, and/or faculty? | | *Yes* | | *No* | | | | *Don’t know/Not sure* |  |
| 1. Are scholarly track residents required to serve as hands-on instructor at a POCUS workshop? | | *Yes* | | *No* | | | | *Don’t know/Not sure* |  |
| **Administration** | | | | | | | | |  |
| 1. Are scholarly track residents required to participate in quality assurance of scans performed in the Emergency Department? | | *Yes* | | *No* | | | | *Don’t know/Not sure* |  |
| 1. Are scholarly track residents required to participate in a POCUS-focused quality improvement project? | | *Yes* | | *No* | | | | *Don’t know/Not sure* |  |
| **Research and Scholarly Achievement** | | | | | | | | |  |
| 1. Are scholarly track residents required to conduct POCUS-focused research? | | *Yes* | | *No* | | | | *Don’t know/Not sure* |  |
| 1. Are scholarly track residents required to attend an ultrasound-focused conference? | | *Yes* | | *No* | | | | *Don’t know/Not sure* |  |
| 1. Are scholarly track residents required to present a POCUS-focused abstract at an ultrasound or emergency medicine conference? | | *Yes* | | *No* | | | | *Don’t know/Not sure* |  |
| 1. How many POCUS-focused abstracts have been presented at ultrasound or emergency medicine conferences by scholarly track residents over the past 3 years (graduating classes of 2018, 2019, and 2020)? | |  | | | | | | |  |
| 1. Are scholarly track residents required to contribute to a POCUS-focused manuscript in a peer-reviewed journal publication? | | *Yes* | | *No* | | | | *Don’t know/Not sure* |  |
| 1. How many POCUS-focused peer-reviewed journal publications have been produced by scholarly track residents over the past 3 years (graduating classes of 2018, 2019, and 2020)? | |  | | | | | | |  |
| **Other** | | | | | | | | |  |
| 1. Do scholarly track residents have dedicated elective time free from clinical shifts to pursue scholarly track goals? | | *Yes* | | *No* | | | | *Don’t know/Not sure* |  |
| If yes, how many weeks are provided over the course of residency? | |  | | | | | | |  |
