# Supplementary material for: National Survey of Point-of-Care Ultrasound Scholarly Tracks in Emergency Medicine Residency Programs
Source: West J Emerg Med. 2021 Aug 21;22(5):1095–101. doi: 10.5811/westjem.2021.5.52118 (PMC8463042; doi:10.5811/westjem.2021.5.52118)
Supplement: Supplementary file 2 [file wjem-22-1095-s002.docx]

**Appendix B: Distribution of Faculty Positions Among Respondents from Unique Residency Programs** (n=199)

| **Position*** | **Number of Respondents (%)** |
| --- | --- |
| Ultrasound Director | 107 (53.8%) |
| Fellowship Director | 62 (31.2%) |
| Residency Director | 49 (24.6%) |
| Ultrasound Resident Education Director | 29 (14.6%) |
| Residency Associate Program Director | 17 (8.5%) |
| Ultrasound Undergraduate Medical Education Director | 14 (7.0%) |
| Ultrasound Research Director | 7 (3.5%) |
| Other Ultrasound Faculty | 6 (3.0%) |

**Responders were allowed to select more than one position*
